# Diabetes, a Contemporary Risk for Parkinson’s Disease: Epidemiological and Cellular Evidences

**DOI:** 10.3389/fnagi.2019.00302

**Published:** 2019-11-08

**Authors:** Domenico Sergi, Justine Renaud, Nicola Simola, Maria-Grazia Martinoli

**Affiliations:** ^1^Nutrition and Health Substantiation Group, Nutrition and Health Program, Health and Biosecurity, Commonwealth Scientific and Industrial Research Organisation (CSIRO), Adelaide, SA, Australia; ^2^Adelaide Medical School, University of Adelaide, Adelaide, SA, Australia; ^3^Cellular Neurobiology, Department of Medical Biology, Université du Québec, Trois-Rivières, QC, Canada; ^4^Department of Biomedical Sciences, University of Cagliari, Cagliari, Italy; ^5^National Institute for Neuroscience (INN), University of Cagliari, Cagliari, Italy; ^6^Department of Psychiatry and Neuroscience, Université Laval and CHU Research Center, Québec, QC, Canada

**Keywords:** dopamine, glucotoxicity, hyperglycemia, mitochondrial dysfunction, nigrostriatal pathway, oxidative stress

## Abstract

Diabetes mellitus (DM), a group of diseases characterized by defective glucose metabolism, is the most widespread metabolic disorder affecting over 400 million adults worldwide. This pathological condition has been implicated in the pathogenesis of a number of central encephalopathies and peripheral neuropathies. In further support of this notion, recent epidemiological evidence suggests a link between DM and Parkinson’s disease (PD), with hyperglycemia emerging as one of the culprits in neurodegeneration involving the nigrostriatal pathway, the neuroanatomical substrate of the motor symptoms affecting parkinsonian patients. Indeed, dopaminergic neurons located in the mesencephalic substantia nigra appear to be particularly vulnerable to oxidative stress and degeneration, likely because of their intrinsic susceptibility to mitochondrial dysfunction, which may represent a direct consequence of hyperglycemia and hyperglycemia-induced oxidative stress. Other pathological pathways induced by increased intracellular glucose levels, including the polyol and the hexosamine pathway as well as the formation of advanced glycation end-products, may all play a pivotal role in mediating the detrimental effects of hyperglycemia on nigral dopaminergic neurons. In this review article, we will examine the epidemiological as well as the molecular and cellular clues supporting the potential susceptibility of nigrostriatal dopaminergic neurons to hyperglycemia.

## Introduction

Since the publication of the pioneering report of Dr. James Parkinson, “An Essay on the Shaking Palsy,” where he described six subjects suffering from a pathological state that he termed *paralysis agitans*, the field of Parkinson’s disease (PD) has phenomenally progressed. In this age of effervescent neuroscientific developments, clinical profiles are more accurately drawn, long-term patient management is improving, and the pursuit of etiological and pathophysiological explanations is starting to bear fruit. The preferential degeneration of the dopaminergic nigrostriatal pathway is well appreciated today, and additional pieces of the puzzle are continuously falling into place. More importantly, the apparent link between diabetes (DM) and PD is rising momentum, with hyperglycemia representing the metabolic culprit linking DM with PD. Even though a handful of medical conditions may cause hyperglycemia, we deliberately hold our focus on DM as it offers a tangible collection of experimental and clinical evidence interlaced with PD pathophysiology.

The present review will lay out the epidemiological evidence linking DM and PD as well as the molecular mechanisms underlying the exacerbated susceptibility of nigrostriatal dopaminergic neurons to hyperglycemia, by illustrating glucose transport and metabolism in the brain and the role of glucotoxicity in promoting neuronal damage and degeneration. The transport kinetics and metabolism of glucose are highly complex, especially in the central nervous system (CNS), and bear significant implications in the initiation of oxidative stress, induction of non-enzymatic glycation and activation of biochemical pathways mediating the effect of glucotoxicity in neurons.

## Parkinson’s Disease and the Vulnerability of the Nigrostriatal Pathway

PD is the prototype of movement disorders. In fact, the most prominent feature of PD is the presence of motor symptoms expressed in a majority of patients as a classical triad of resting tremor, bradykinesia, and rigidity (Obeso et al., 2000). Non-dopaminergic (extranigral) motor features may also arise and result in falls, freezing of gait, speech impairment and difficulty in swallowing. Accompanying motor symptoms are equally disabling non-motor manifestations, such as psychiatric disturbances, dementia and autonomic failure (Schapira et al., 2017).

The neuropathological hallmark of PD is the progressive and relentless degeneration of dopaminergic neurons located in the substantia nigra *pars compacta*. Idiopathic PD still composes approximately 90–95% of diagnoses that are not always faithfully represented by genetic forms in terms of symptomatology and pathophysiology (Houlden and Singleton, 2012).

In the mid-1900s, the role of the substantia nigra *pars compacta* in PD was strengthened by findings enlisting dopamine (DA) as a key contributor to movement performance (Carlsson et al., 1958; Ehringer and Hornykiewicz, 1960) and demonstrating the projection of nigral neurons to the dorsal striatum (Hornykiewicz, 1966). Another significant leap forward in the field of PD was the identification of the first gene mutation, explicitly a single-point mutation in the SNCA gene coding for α-synuclein, found to cause a dominant familial form of early-onset PD (Polymeropoulos et al., 1997). This finding triggered a sequence of discoveries concerning genetic forms of the pathology. Disease-causing duplications (Chartier-Harlin et al., 2004) and triplications (Singleton et al., 2003) of the SNCA gene were identified, and other genes were added in subsequent years (Obeso et al., 2017).

The discovery of 1-methyl-4-phenyl-1,2,3,6-tetrahydropyridine (MPTP) as a mitochondria-targeting agent of parkinsonism, broke new ground for the exploration of other chemicals, such as pesticides (Langston et al., 1983; Priyadarshi et al., 2000; Greenamyre et al., 2001; Furlong et al., 2015), solvents (Goldman et al., 2012; Pezzoli and Cereda, 2013), metals (Gorell et al., 1997; Finkelstein and Jerrett, 2007) and organochlorines (Steenland et al., 2006; Weisskopf et al., 2012), subsequently shown to constitute risk factors acting *via* bioenergetic disruption and oxidative mechanisms. Elements of infection were also suggested long ago to promote PD (Charcot, 1877; Gowers, 1886), substantiated by the occurrence of postencephalitic parkinsonism that succeeded the great pandemic of *encephalitis lethargica* in the early 1900s (Schwab et al., 1956; Vilensky et al., 2010). Later, studies found certain of these exogenous elements to represent a greater hazard in individuals carrying a specific genetic profile. Indeed, a host of genetic variants is known today to synergistically increase the odds of developing PD, in gene-environment interactions but also as standalone risk factors. For example, p.G2385R in the LRRK2 gene is common among Chinese and Japanese populations and approximately doubles the risk for PD (Bonifati, 2007), while the REP1 microsatellite marker of the SNCA promoter region was consistently associated with a 1.4-fold increased risk of PD (for a review, see Warner and Schapira, 2003; Polito et al., 2016).

Aside from these genetic and environmental factors specific to certain individuals, the substantia nigra* pars compacta* appears to be relatively susceptible to aging outside of a pathological context (Rudow et al., 2008; Buchman et al., 2012; Reeve et al., 2014), as spontaneous cell loss occurs at an estimated rate of 4.7–9.8% per decade in humans (Fearnley and Lees, 1991; Ma et al., 1999). Moreover, iron contents (Bilgic et al., 2012), levels of iron-binding neuromelanin (Double et al., 2003), neuroinflammation (Calabrese et al., 2018), oxidative stress-induced mitochondrial DNA deletions (Reeve et al., 2014), and malfunction of the ubiquitin-proteasome system and autophagy clearance pathways (Li and Li, 2011; Rubinsztein et al., 2011; Jana, 2012) were all shown to increase drastically with age, especially so in the substantia nigra *pars compacta* (Dexter et al., 1989; Corral-Debrinski et al., 1992; Soong et al., 1992; Fedorow et al., 2006; Daugherty and Raz, 2013). Coherently, in the setting of PD, nigrostriatal neurons that contain more neuromelanin are at higher risk of degenerating (Marsden, 1983; Hirsch et al., 1988; Gibb and Lees, 1991; Kastner et al., 1992; Zecca et al., 2001), microgliosis is relatively more pronounced in the substantia nigra* pars compacta* (McGeer et al., 1988; Lawson et al., 1990; Kim et al., 2000), nigral mitochondrial DNA deletions accumulate (Bender et al., 2006, 2008) and cellular clearance pathways are impaired (Cuervo et al., 2010; Dehay et al., 2010).

Despite their apparent disparity, multiple mechanisms converge to cause the degeneration of nigrostriatal neurons and possibly contribute to oxidative stress to which these neurons may be more vulnerable (Surmeier, 2007; Brichta and Greengard, 2014; Haddad and Nakamura, 2015; Oliveira et al., 2017; Surmeier et al., 2017). This concept dates back to the 1980s (Lenzi et al., 1979; Youdim et al., 1989; Götz et al., 1990; Jenner, 1991), but its popularity has waxed and waned at the rhythm of discoveries; nevertheless, the idea that metabolic oxidative insults may underlie the specific vulnerability of nigrostriatal neurons has lately gained momentum (Obeso et al., 2017).

It is critical that nigrostriatal neurons distinguish themselves from other dopaminergic neurons, especially their mesocorticolimbic analogs, as they all share the liability of employing dopamine as a neurotransmitter, recognized to generate reactive oxygen species (ROS) upon auto-oxidation (Segura-Aguilar et al., 2014) and to require ROS-producing monoamine oxidases for its degradation (Graham, 1984; Andersen et al., 1994). In this respect, we argue that nigrostriatal dopaminergic neurons display the following phenotypic idiosyncrasies that may underlie their vulnerability to oxidative stress instigated by genetic mutations, environmental toxins, aging and combinations of these factors:

1. They are embedded in a particular environment, the substantia nigra *pars compacta*, which stores considerable amounts of iron ions (Hirsch and Faucheux, 1998; Chinta and Andersen, 2008) accumulating with age (Daugherty and Raz, 2013) and known to participate in deleterious Fenton reactions with hydrogen peroxide to produce the very reactive hydroxyl radical (Youdim et al., 1989).
2. They express lower levels of vesicular monoamine transporter 2 (VMAT2) than most other catecholaminergic neurons, implying the protracted presence of dopamine in the cytosol leading to the inevitable formation of toxic oxidative by-products and, ultimately, potentially toxic neuromelanin (Liang et al., 2004).
3. Despite constituting a very small cluster of cells (German et al., 1988; Percheron et al., 1989), they are required to innervate a very large surface, the dorsal striatum, which entails tremendous branching of axons harboring numerous vesicular release sites ridden with potentially pathogenic α-synuclein (Parent and Parent, 2006; Matsuda et al., 2009);
4. To enable corticostriatal circuits, they constitute the greatest striatal input of uninterrupted tonic dopamine relying on autonomous pacemaking activity at the source of high baseline levels of calcium ions, which enhance oxidative phosphorylation, mitochondrial membrane hyperpolarization and superoxide anion production (Guzman et al., 2010; Pacelli et al., 2015).
5. To maintain their neurotransmission activities and drawn-out axonal harbors, nigral neurons keep an army of mitochondria to fulfill their exorbitant energetic needs, also implying elevated basal rates of oxidative phosphorylation and greater production of electron transport chain superoxide anion by-products (Pacelli et al., 2015).
6. Last, they are endowed with a limited calcium-buffering capacity (Foehring et al., 2009) and scanty endogenous antioxidative defenses (Bharath et al., 2002), consequent of their low expression of calbindin (Iacopino and Christakos, 1990; Yamada et al., 1990; Liang et al., 1996; Dopeso-Reyes et al., 2014) and glutathione (Kang et al., 1999), respectively.

These features unite to augment the basal oxidative burden to which nigrostriatal neurons are subjected. Upon further exposure to environmental or genetic insults, endogenous antioxidative defenses are eventually overwhelmed: this turning point typified by the failure of neurons to cope with the added oxidative load constitutes the origin of oxidative stress. Sustained in time and enhanced by aging processes, oxidative stress may eventually lead to the death of nigrostriatal neurons featuring PD. Following this premise, studies have employed primary neuron cultures (Pacelli et al., 2015), rat brain slices (Carbone et al., 2017), rodents (Vidyadhara et al., 2016) and primate (Dopeso-Reyes et al., 2014) models to demonstrate that nigrostriatal dopaminergic neurons are more vulnerable to neurotoxins that induce PD-like phenotypes in comparison to other neuronal populations, exemplarily dopaminergic neurons lodged in the neighboring ventral tegmental area.

Other sources of oxidative stress that are not specific to PD may yield similar results and shore up the idea of the selective vulnerability of nigrostriatal dopaminergic neurons. Indications of this are provided by epidemiological evidence showing a higher occurrence of PD in patients suffering from other pathologies with a significant component of oxidative stress, such as DM (Cereda et al., 2012; Sun et al., 2012; Santiago and Potashkin, 2013), ischemic stroke (Huang et al., 2013) and sleep apnea (Snyder and Cunningham, 2018). Interestingly, PD is significantly less frequent in patients with the hyperuricemic illness gout wherein the antioxidative effects of uric acid may, in fact, protect nigrostriatal neurons (Alonso and Sovell, 2010; Huang et al., 2017). Recently, our group has provided robust evidence for the selective demise of nigrostriatal neurons compared to mesocorticolimbic neurons over the course of 6 months of sustained hyperglycemia, attended by alterations in glial profiles that indirectly supported a role for oxidative stress as a key conspirator in neuronal death (Renaud et al., 2018), further evinced by *in vitro* groundwork (Bournival et al., 2012; Renaud et al., 2014). Notably, despite modest neurodegeneration, hyperglycemia evoked motor deficits strikingly reminiscent of parkinsonian motor symptoms.

## Epidemiological Evidence: Parkinson’s Disease in Diabetic Patients

The first indications of a possible association between DM and PD date back to almost 60 years ago. Studies conducted in parallel showed that DM exacerbates the progression of motor and cognitive deficits in PD (Schwab, 1960), and that non-diabetic parkinsonian patients present impaired glucose tolerance (Barbeau et al., 1961) and hyperglycemia (Boyd et al., 1971). Since then, these findings have been reiterated in parkinsonian patients medicated with dopamine-replacing therapy (Lipman et al., 1974; Sandyk, 1993; Cereda et al., 2012), but also in drug-naïve parkinsonian patients who still displayed higher-than-normal levels of fasting blood glucose, within the prediabetic diagnostic range (Santiago and Potashkin, 2015). In addition, dietary habits featuring an elevated intake of high glycemic indexed carbohydrates have more recently been associated with greater odds of developing PD (Cheng et al., 2009; Murakami et al., 2010; Yang and Cheng, 2010; Okubo et al., 2012; Stafstrom and Rho, 2012).

In light of accumulating reports showing a possible association between DM and PD, numerous prospective cohort studies were elaborated to unveil their temporal and cause-effect relationship (Wirdefeldt et al., 2011; Santiago and Potashkin, 2013), with most of these studies reporting pre-existing DM as a risk factor for the development of PD (Arvanitakis et al., 2007; Hu et al., 2007; Becker et al., 2008; Driver et al., 2008; Cereda et al., 2011; Xu et al., 2011; Cereda et al., 2012). To dispel any doubt, one group addressed the question by quantifying comorbidity of DM in almost 40,000 single clinical measurements and robustly confirmed the relationship between DM and increased risk for the development of PD later in life (Klimek et al., 2015). Moreover, other studies provided evidence supporting a possible link between DM and the severity of PD-related symptoms (Schwab, 1960; Cereda et al., 2012). Likewise, in older individuals without PD who displayed parkinsonian-like signs, like gait disturbances, the severity of these signs was associated with existing DM (Arvanitakis et al., 2007).

In consideration of the close relationship between DM and PD pathogenesis, anti-diabetic drugs are emerging as strong candidates for the development of novel promising therapeutic approaches for PD. A sharp interest in these substances surfaced following reports evoking a decreased risk of developing PD in diabetic patients employing anti-hyperglycemic drugs. Specifically, the incidence of PD was found to be lower in diabetic patients treated with metformin or thiazolidinediones, which are widely used glucose-lowering medications (Wahlqvist et al., 2012; Brauer et al., 2015). With this regard, current clinical trials are investigating the anti-diabetic and glucose-lowering effect of exenatide, a glucagon-like peptide 1 (GLP-1) receptor agonist acting as an incretin mimetic and increasing insulin secretion from the pancreatic beta cells of the islets of Langerhans. Moreover, activation of the GLP-1 receptor induces the activation of the PI3K/AKT and RAS-extracellular signal-related kinase (ERK) pathways which, as described below, are known to mediate the biological effects of insulin (Athauda and Foltynie, 2018). Furthermore, the stimulation of the GLP-1 receptor is able to improve insulin resistance and restore the insulin-mediated functions that become impaired in PD thereby acting as a neuroprotective factor, inhibiting apoptosis, inflammation and oxidative stress (Athauda and Foltynie, 2016a,b). An initial open-label trial carried out in 44 parkinsonian patients receiving dopamine-replacing therapy showed that twice-daily injections of exenatide for 12 months improved overnight off-medication motor symptoms and dementia, rated according to the unified PD rating scale and the Mattis dementia rating scale, respectively (Aviles-Olmos et al., 2013). This trial employed a washout design wherein exenatide treatments were ceased at 12 months and patients were re-evaluated at later time points. Remarkably, the benefits observed at 12 months still held at 14 (Aviles-Olmos et al., 2013) and 24 months (Aviles-Olmos et al., 2014). This encouraged the elaboration of subsequent double-blinded, placebo-controlled trials once again designed to include a washout period and to measure symptoms in off-medication patients. The benefits of exenatide were reiterated and supplementary DaTscan neuroimaging results, which conveyed information on the amount of presynaptic dopamine transporter (DAT) left in the striatum of patients, confirmed a significant positive difference between treated and control parkinsonian patients (Athauda et al., 2017). The link between DM and PD is further strengthened by the putative therapeutic potential of metformin in PD. Indeed, besides its well-established role in the treatment of type II DM, metformin is emerging for its ability to counteract neurodegenerative diseases by modulating intracellular pathways impinging on neuronal longevity (Rotermund et al., 2018). Particularly, metformin has been shown to exert beneficial effects on neuronal mitochondria (Fitzgerald et al., 2017) whose dysfunction has been described as a pivotal defect in the pathogenesis of PD (Haddad and Nakamura, 2015). Furthermore, metformin is able to modulate neuronal energy metabolism by promoting a mild inhibition of mitochondrial complex 1 and leading to a decrease in oxidative stress and the activation of AMP-activated protein kinase (AMPK; Rotermund et al., 2018) which, beside acting as a central intracellular energy sensor, may exert neuroprotective effects (Ng et al., 2012; Bobela et al., 2017). Despite from a mechanistic point of view metformin may represent a valuable disease-modifying tool, its therapeutic potential in PD remains to be fully investigated. Indeed, animal studies aimed at investigating the effect of metformin on PD remain controversial, with some (Patil et al., 2014; Bayliss et al., 2016; Lu et al., 2016; Katila et al., 2017) but not all reporting a neuroprotective effect of metformin against MPTP-induced dopaminergic neuron damage (Ismaiel et al., 2016). Importantly both metformin and MPTP target the mitochondrial complex 1 and their potential interaction at this level may explain the discrepancies among different studies. In regard to human studies, clinical data on the effect of metformin of PD are lacking with the majority of the studies conducted so far comparing metformin against or using it in combination with other anti-hyperglycemic drugs, making it difficult to identify a clear-cut effect of metformin on PD progression (Rotermund et al., 2018). Furthermore, there is also evidence that metformin may increase the risk of developing PD (Kuan et al., 2017), but this remains to be fully elucidated and further studies are warranted to confirm this relationship.

Despite the recent medical advances in teasing out the cause-effect relationship linking DM and PD, it is still unclear which of the multifarious metabolic changes that occur in DM trigger the nigrostriatal degeneration underlying PD. The two predominant lines of thought grant importance to the dimensions of defective insulin signaling and oxidative stress.

## Diabetes, a Current Epidemic

DM is the most widespread chronic metabolic disorder, estimated to affect over 400 million adults worldwide, with the prevalence of type II DM having doubled since 1980 on the likely account of the marked rise in overweight and obese phenotypes in the population (World Health Organization, 2016). Today, DM has joined the infamous list of leading causes of death, responsible for 8.4% of global mortality in adulthood (International Diabetes Federation, 2013). Both types I and II DM occur from the progressive failure of the body to manage circulating blood glucose. However, the etiopathogenesis of type I DM (5–10% of diabetic patients) is specifically rooted in the autoimmune destruction of insulin-producing pancreatic β cells and usually, but not always, develops before adulthood (Yoon and Jun, 2001). On the other hand, type II DM ensues from peripheral resistance to and deficient production of insulin, alongside other secondary metabolic elements. Type II DM is more frequently diagnosed in adults although currently, youth-onset forms are dramatically on the rise (Correia et al., 2008; Nadeau et al., 2016; Sergi et al., 2019). In light of its relative or absolute absence, insulin fails to promote glucose transporter 4 (GLUT4)-dependent glucose uptake in skeletal muscle and adipose tissue, to inhibit hepatic glucose production and to regulate peripheral glucose homeostasis by acting directly on the brain which, in turn, relies on insulin signaling to control peripheral glucose metabolism (Tups et al., 2017; Sergi et al., 2019). The defective insulin signaling culminates in chronically sustained hyperglycemia and impaired postprandial glucose disposal, synonymous of glucose intolerance (for a detailed review, see Zaccardi et al., 2016).

Granted the multiple aspects of health it impinges on, DM nurtures the development of a myriad of diverse comorbidities, all having hyperglycemia as a common denominator. Indeed, DM has been associated with a variety of microvascular and macrovascular complications including retinopathies, nephropathies, coronary heart disease (Eppens et al., 2006; Pambianco et al., 2006; Gregg et al., 2016). However, for the purpose of this review only data relative to the effect of diabetic hyperglycemia on the nervous system will be examined. Indeed, the nervous system is not exempt from the deleterious effect of DM with neuropathies remaining the most prevalent neuronal affections in this metabolic disease. Peripheral nerves are more susceptible to the detrimental effects of hyperglycemia as they are devoid of blood-brain barrier protection, which may limit exposure to high circulating glucose levels (Bansal et al., 2006; Rajabally et al., 2017). Nonetheless, the protective effect of the blood-brain barrier against hyperglycemia has been challenged (Jacob et al., 2002). In agreement with this paradigm, the complications of DM also affect the CNS (Biessels et al., 2002) with hyperglycemia standing as a risk factor for PD (Pagano et al., 2018).

## Glucotoxicity: Physiological Considerations

The brain accounts for approximately 2% of total body weight, nevertheless it requires up to 20% of total oxygen and energy consumed by the body (Raichle and Gusnard, 2002). Although neurons can use different energetic substrates, including lactate, pyruvate, ketone bodies, glutamate, glutamine, and aspartate (van Hall et al., 2009; Amaral, 2013; Lutas and Yellen, 2013), glucose represents their preferred metabolic substrate (Simpson et al., 2007, 2008). Unlike other energy-greedy cells endowed with glycogen stores, like striated muscle cells, neurons cannot call upon glycogenolysis for sustenance, as glycogen is almost exclusively confined to astrocytes in the brain (Watanabe and Passonneau, 1973; Sagar et al., 1987; Dienel and Cruz, 2006, 2015). Intraneuronal glucose is thus mainly provided from the extracellular environment, which itself is supplied by the circulation. This implies the need for glucose to cross several barriers whose transport kinetics assuredly influence its metabolism in neurons.

Glucose transporters (GLUTs) ubiquitously present at the surface of cells accomplish the function of taking up glucose from the extracellular environment. In peripheral tissues, such as the skeletal muscle and adipose tissue, glucose uptake is mediated by GLUT4, an insulin-dependent glucose transporter. On the contrary, in the vast majority of the CNS glucose transport is mediated by insulin-independent GLUTs (James et al., 1988; Huang and Czech, 2007). GLUT1, one of the insulin-independent GLUT, is particularly abundant in the brain microvasculature, distributed with a 1:4 ratio between the luminal and abluminal surfaces of the endothelium, and plays a pivotal role in mediating glucose transport across the blood-brain barrier (Maher et al., 1994; Simpson et al., 2001, 2008). GLUT1 is also the main transporter employed by glial cells, especially astrocytes (Dick et al., 1984; Gerhart et al., 1989; Maher et al., 1994; Simpson et al., 2001). Neurons, on the contrary, overwhelmingly employ the insulin-independent GLUT3 isoform densely located on the cell surface but more intensely so at the neuropil (Simpson et al., 2008). Thus, neuronal glucose uptake can be defined as an insulin-independent two-step process. Circulating glucose must first penetrate the endothelium and astrocytic endfeet of the blood-brain barrier *via* GLUT1 and subsequently be taken up by neurons in a GLUT3-dependent fashion.

Considering that GLUTs are facilitative, bidirectional, energy-independent transporters, they do not allow for glucose accumulation but for its equilibration between compartments in a manner that smoothens concentration gradients. Thus, the characteristic kinetics of brain GLUTs, rather than insulin, is the driver of glucose uptake into brain cells. Brain GLUTs possess variable degrees of affinity for glucose, with the highest being reported for neuronal GLUT3 (*K*_m_ ~1.5 mM) which allows this transporter to work efficiently even at glucose concentrations surrounding the neurons in the order of 1–2 mM (Colville et al., 1993; Maher et al., 1996). In fact, GLUT3 also presents the greatest turnover for glucose, defined as the number of transport cycles per transporter per second (Rumsey et al., 1997; Manolescu et al., 2007) and considering its abundance in neurons provides them with preferential access to glucose (Simpson et al., 2008). Thus, in light of the kinetics of this transporter, glycemia must drop significantly before neuronal glucopenia manifests (Rao et al., 2006). However, under these circumstances, the brain can call upon different metabolic pathways and harvest energy from substrates other than glucose, as already discussed (van Hall et al., 2009; Amaral, 2013; Lutas and Yellen, 2013).

According to computational studies performed by Simpson et al. (2007), neurons and astrocytes only contain ~0.9 mM of glucose in normoglycemic steady-state conditions. Similarly, brain extracellular concentrations of glucose merely reach ~1.4 mM. Even endothelial cells that express GLUT1 and are in direct contact with blood-borne solutes display concentrations of ~3.8 mM. This discrepancy between expected compartment equilibration and markedly lower levels of glucose in brain parenchymal and endothelial cells may be explained by transport inhibition at the level of the blood-brain barrier. Indeed, endothelial GLUT1 undergoes allosteric inactivation by adenosine triphosphate (ATP) indicating that its activity is a direct function of energy availability (Lowe and Walmsley, 1986; Carruthers and Helgerson, 1989; Levine et al., 1998). Consequently, when glucose levels suffice brain energy demand its uptake by endothelial cells is inhibited and glucose supply to brain parenchyma decreases. Conversely, when glycolytic demands increase and ATP levels become depleted, GLUT1 activity is uninhibited and glucose may efficiently be transferred to surrounding tissues (Carruthers, 1986; Cloherty et al., 1996).

In opposition to the hypoglycemia-induced compensatory upregulation of brain GLUTs and metabolic switch to alternative substrates to meet brain energy requirements, hyperglycemia, as demonstrated in rodent models, does not trigger protective mechanisms aimed at limiting excess glucose entering the brain (Nagamatsu et al., 1994; Simpson et al., 1999; Santiago et al., 2006; Anitha et al., 2012). In support of this, in rodent models of sustained DM-induced hyperglycemia, intracerebral glucose concentrations are also augmented (Jacob et al., 2002; de Vries et al., 2003; Gomez and Barros, 2003; McCrimmon et al., 2003), which argues against the protective role of the blood-brain barrier against hyperglycemia and reinforces the susceptibility of the brain to chronically elevated circulating glucose levels. However, these experiments were performed on whole brain tissues and reliable intraneuronal measurements are still lacking. Nevertheless, computational renderings taking into account GLUT kinetics and their distribution predict greater glucose permeation in all brain regions under hyperglycemia (Simpson et al., 2007). In further support of this notion, our group has recently demonstrated that intracellular as well as extracellular glucose concentrations are increased in midbrain and striatum during long-term hyperglycemia in rats (Renaud et al., 2018). Thus, we can faithfully support the idea that hyperglycemia causes an upwelling of intraneuronal glucose likely to hold pathological implications for cellular fitness. Moreover, in humans, brain glucose uptake was reported to be inversely correlated with insulin sensitivity and positively associated with body mass index (BMI) as well as signs of inflammation in the hypothalamus (Rebelos et al., 2018). Thus, hypothalamic inflammation, may also occur in humans and induce insulin resistance leading to dysregulation of glucose homeostasis (Tups et al., 2017). This provides further evidence for the physiological relevance of the relationship between impaired glucose metabolism, insulin resistance, DM and enhanced brain glucose uptake.

## Insulin and Neuronal Homeostasis

Although insulin occupies an increasingly acknowledged role in the human CNS, by activating insulin-responsive neurons responsible for controlling appetite, peripheral glucose homeostasis, reward, cognition and memory (Craft et al., 1996; Benedict et al., 2004, 2007; Anthony et al., 2006; Hallschmid et al., 2008; Reger et al., 2008; Khanh et al., 2014; Tups et al., 2017), this peptide hormone is not required for glucose transport in the brain parenchyma or in most brain resident cells (Tups et al., 2017). Nevertheless, besides the aforementioned biological effects in the CNS, insulin is also known to regulate physiological functions independent from and beyond the regulation of glucose homeostasis, including neuronal development, differentiation, plasticity and survival (Bassil et al., 2014). In physiological conditions, insulin concentrations are highest in the pons, medulla and hypothalamus, and lowest in the occipital cortex and thalamus (Banks and Kastin, 1998), with the midbrain, affected in PD, falling within the average. In agreement with the widespread effect of insulin in the brain, its receptor is also widely expressed throughout the CNS (Havrankova et al., 1978a; Hill et al., 1986; Gralle, 2017), and possesses similar kinetic properties to its peripheral counterpart (LeRoith et al., 1988; Zahniser et al., 1984). Insulin signal transduction pathway in neurons, similarly to peripheral cells, relies on the activation of PI3K/AKT and RAS-ERK pathways which are responsible for the stimulation of synaptic plasticity, inhibition of neuronal apoptosis and gene transcription (Athauda and Foltynie, 2016a). In light of its ability to activate pathways that promote survival, neurogenesis, and inhibition of apoptosis, insulin is generally accepted to act as a neurotrophic factor. In support of this, insulin has been shown to counteract the neurotoxic effect exerted by excitotoxicity or oxidative stress *in vitro*, confirming its neuroprotective role (Kim and Han, 2005; Duarte et al., 2006; Sun et al., 2010; Ribeiro et al., 2014). *In vivo*, near-direct application of insulin on the CNS, *via* intranasal or intracerebroventricular treatments, are highly effective in promoting various aspects of neuronal health, as demonstrated in a rat model of PD (Haas et al., 2016; Pang et al., 2016).

Studies attempting to demonstrate that neuronal death directly ensues from hypoinsulinemia employed peripheral insulin administration to reverse cellular or structural damage induced by streptozotocin treatments in the CNS of rodents (Ramanathan et al., 1999; Moreira et al., 2005, 2006; Hung et al., 2014). However, they do not take into account the various peripheral benefits exerted by insulin replacement therapy, and specifically improved glycemic control. Such strategies exploiting organism-wide paradigms to establish causality between hypoinsulinemia and CNS neuronal death are based on the misconception that intracerebral insulin is solely provided from the circulation, itself supplied by the pancreas. As a matter of fact, insulin concentrations are considerably greater in the brain parenchyma than in the plasma, and are unexpectedly upregulated in hypoinsulinemic, hyperglycemic streptozotocin-treated rats (Havrankova et al., 1978a, b, 1979; Gupta et al., 1992; Banks et al., 1997b). In support of this, the insulin receptor is downregulated in peripheral tissues but remains unaffected in the brain of streptozotocin models (Pacold and Blackard, 1979; Pezzino et al., 1996). These preliminary findings led to the discovery that, besides being taken up through the blood-brain barrier (Margolis and Altszuler, 1967; Woods and Porte, 1977; Banks et al., 1997a), insulin is synthesized by neurons within the mammalian brain (Dorn et al., 1983; Birch et al., 1984; Young, 1986; Deltour et al., 1993; Devaskar et al., 1994; Schechter et al., 1994; Frölich et al., 1998; Mehran et al., 2012; Molnár et al., 2014). Importantly, one group demonstrated that hyperglycemia enhanced insulin production by cortical neurons (Molnár et al., 2014), which may be a mechanism to maintain appropriate intracerebral concentrations of insulin when pancreatic supplies plunge (Havrankova et al., 1979; Gupta et al., 1992; Banks et al., 1997b). Thus, hypoinsulinemia does not seem to be the metabolic culprit linking type I DM and PD, while hyperglycemia may represent a more plausible mediator bridging the gap between DM and PD. Nonetheless, we cannot overlook the fact that insulin resistance is the main feature of type II DM and that insulin resistance also occurs in the brain (Athauda and Foltynie, 2016a) with defective insulin signaling being identified as an important player in the pathogenesis of neurodegenerative disorders and particularly Alzheimer’s disease (AD; Steen et al., 2005; Talbot et al., 2012; Freiherr et al., 2013; Arnold et al., 2018). Besides its well-established role in AD, emerging evidence suggests insulin resistance also occurring in the brain of PD patients pointing to impaired brain insulin signaling as a potential contributing factor to the pathological defects of PD (Athauda and Foltynie, 2016a). In support of this, a downregulation of the insulin receptor in the substantia nigra *pars compacta* and increased insulin resistance has been described in patients affected by PD (Moroo et al., 1994; Takahashi et al., 1996; Duarte et al., 2012), a phenomenon which may occur before the death of dopaminergic neurons (Moroo et al., 1994). Particularly, AKT/protein kinase β appears to be a critical node in mediating the physiological effect of insulin, and impaired AKT signaling, as described in insulin-resistant individuals, may be involved in the pathogenesis of PD, further supporting its association with insulin resistance (Greene et al., 2011). In agreement with this, AKT phosphorylation has been reported to be decreased in the brain of PD patients following post mortem analysis (Malagelada et al., 2008). Moreover, inhibition of AKT signaling promotes dopaminergic cell death (Xu et al., 2014) and loss of dopaminergic phenotype in axons (Kim et al., 2011), providing a further mechanistic link between impaired insulin signaling and PD (Canal et al., 2014). Thus, the compromised neurotrophic action of insulin, due to defective insulin signaling, appears to be at the interphase between insulin resistance and PD pathogenesis. Nonetheless, other putative mechanisms may link insulin resistance and PD, including α-synuclein aggregation and toxicity, defects in mitochondrial function and neuroinflammation (Athauda and Foltynie, 2016a). Indeed, the activation of insulin signaling can modulate the degradation of alpha-synuclein and inhibit alpha-synuclein fibril formation by activating the insulin-degrading enzyme (Sharma et al., 2015) which is supported by the fact that reversing insulin resistance prevented alpha-synuclein-induced toxicity (Kao, 2009). In regard to the role of insulin in modulating mitochondrial function, activation of insulin signal transduction pathway and particularly PI3K/AKT plays a key role in maintain NAD^+^/NADH ratio which, in turn, regulates mitochondrial biogenesis and function by modulating the sirtuin 1/proliferator-activated receptor-gamma coactivator 1-α (SIRT/PGC-1α) axis (Cheng et al., 2010). Not surprisingly, animal models of insulin resistance display a decrease in mitochondrial proteins in the substantia nigra (Khang et al., 2015). Despite it is tempting to blame insulin resistance as the driver on neuronal mitochondrial disfunction, this remains elusive also in consideration of the fact that mitochondrial dysfunction itself may cause, and therefore precede, the onset of insulin resistance. Finally, insulin signaling may directly interfere with inflammation by upregulating IκBα and therefore inhibiting kappa-light-chain-enhancer of activated B cells (NF-κB) which is pivotal in regulating the expression of pro-inflammatory genes and has been implicated in PD neuro-inflammation (Kirkley et al., 2019).

## Cellular Evidence: Neuronal Oxidative Stress in Hyperglycemia

The clinical significance of hyperglycemia emerged upon its formal identification as the prime culprit in comorbid diabetic complications (Nathan et al., 1993; UK Prospective Diabetes Study (UKPDS) Group, 1998; Gaede et al., 2008; Giacco and Brownlee, 2010). The most affected collateral targets in DM are the kidneys, the cardiovascular system and the nervous system (Nathan, 1993). Despite their many functional and phenotypic differences, the cells of these systems share the distinct liability of taking up glucose in an extracellular concentration-dependent but insulin-independent manner (Kaiser et al., 1993; Heilig et al., 1995). Indeed, mesangial and endothelial cells predominantly express the insulin-independent glucose transporter GLUT1 (Sone et al., 2000; Brosius and Heilig, 2005; Mueckler and Thorens, 2013); likewise, brain cells express the insulin-independent GLUTs, GLUT1 and GLUT3 with the latter being particularly abundant in neurons (Mantych et al., 1992; Nagamatsu et al., 1993; Simpson et al., 2008). Although it is clear that glucose concentrations rise in these cells under conditions of hyperglycemia, how exactly glucotoxicity-mediated cellular damage occurs is not as forthright. The current consensus is that oxidative stress may represent the principal mediator of hyperglycemia-induced diabetic complications (Giugliano et al., 1996; Ceriello, 2003; Brownlee, 2005; Araki and Nishikawa, 2010).

Owing to their steep demands in ATP, neurons possess a high basal oxidative metabolism compared to other types of cells in the brain or elsewhere and are thus plausibly more vulnerable to additional sources of stress. Although the literature does not provide an adequate account of the mechanisms underlying hyperglycemia-induced oxidative stress in the CNS, and most importantly in nigrostriatal dopaminergic neurons, we can speculate on the events that contribute to this critical coping threshold based on experimental data and our knowledge of neuronal glucose metabolism.

### Mitochondrial Mechanisms

**Figure 1:**
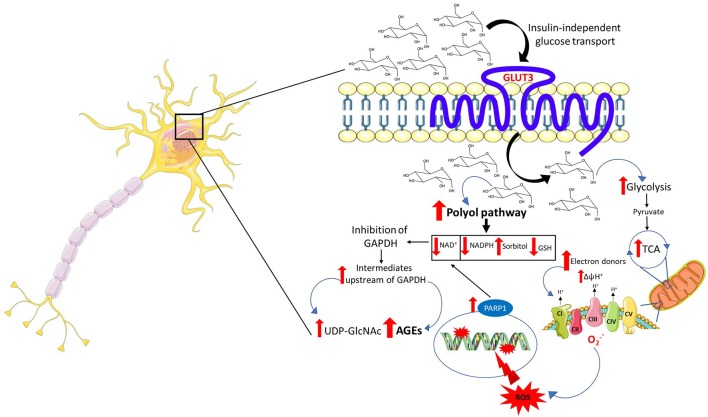
Mechanisms of neuronal glucotoxicity. In the early stages of hyperglycemia, there is an upregulation of glucose flux through glycolysis and the tricarboxylic acid cycle (TCA). This results in enhanced electron donor supply to the mitochondrial electron transport chain, leading to an increase in the proton gradient across the mitochondrial inner membrane which promotes the formation of reactive oxygen species (ROS). ROS-induced DNA damage triggers the activation of the NAD^+^-dependent DNA repair enzyme, poly (adenosine diphosphate-ribose) polymerase 1 (PARP1), responsible for depleting neuronal NAD^+^. The increase in intracellular glucose activates the polyol pathway which, in concert with PARP1, reduces intracellular NAD^+^ inhibiting glyceraldehyde triphosphate dehydrogenase (GAPDH) and therefore glycolysis. The inhibition of GAPDH provokes the accumulation of upstream triose phosphates then converted to glycating agents, thereby promoting the formation of advanced glycation end products (AGEs). The accumulation of fructose, another metabolite upstream GAPDH, triggers the activation of the hexosamine pathway and the subsequent build-up of UDP-N-acetylglucosamine (UDP-GlcNAc). Furthermore, the activation of the polyol pathway depletes NADPH and, alongside mitochondrial ROS, contributes to oxidative stress in light of its role as a cofactor for glutathione reductase, which, in turn, is crucial in maintaining the intracellular pool of reduced glutathione.

Following an upsurge in intracerebral and intraneuronal glucose, a plausible event that may occur is an amplified flux of electron donors toward oxidative phosphorylation. In a first scenario, high intraneuronal glucose concentrations may provide the glycolytic pathway and the tricarboxylic acid cycle (TCA) with more fuel, culminating in increased production of electron donors which are funneled toward the electron transport chain to fuel oxidative phosphorylation. In a second scenario, taking into account the possibility of an astrocyte-neuron lactate shuttle in hyperglycemia, lactate derived from glycolysis in astrocytes may enter the neurons and be converted to pyruvate to power the TCA. The end result is electron transport chain overload and subsequent increase in the proton gradient across the mitochondrial inner membrane which, in turn, impairs the transport of electrons across the mitochondrial complexes favoring the leakage of electrons and the formation of ROS (Figure 1).

ROS induce cellular damage by targeting macromolecules, namely protein, lipids, and DNA. Particularly, oxidative stress-induced DNA damage results in strand breaks leading to the activation of the NAD^+^-dependent DNA repair enzyme, poly (adenosine diphosphate-ribose) polymerase (PARP1), which, in turn, by depleting intracellular NAD^+^, in concert with the polyol pathway, inhibits glyceraldehyde triphosphate dehydrogenase (GAPDH; Du et al., 2003; Figure 1). The inhibition of GAPDH represents a critical node in promoting glucotoxicity. Indeed, its inhibition disrupts intracellular glucose metabolism, promotes the buildup of metabolites acting as precursors of the advanced glycation end products (AGEs) and is responsible for the activation of the hexosamine pathway. Generation of ROS contributes to a self-perpetuating cycle of DNA damage and consequent PARP1 activation, which in concert with the polyol pathway inhibits GAPDH, leading to a permanent glycolytic impasse and sustained oxidative stress (Brownlee, 2005).

It is important to note that the key results in support of Brownlee’s theory were obtained in bovine aortic endothelial cells treated with 30 mM of glucose for 7 days (Nishikawa et al., 2000; Du et al., 2001). Consequently, these conditions may not reflect the pathophysiological mechanisms taking place in the brain of animals exposed to high levels of glucose. Indeed, a small body of literature investigating these mechanisms in the CNS and the periphery of rodents does not clearly validate this model in neurons (Thurston et al., 1995; Kaur and Bhardwaj, 1998; Thakran and Baquer, 2003; Price et al., 2006; Chowdhury et al., 2010; Akude et al., 2011; Hinder et al., 2013; Zheng et al., 2017). Despite, mitochondria appearing to be involved in perpetuating the effect of hyperglycemia by producing ROS, the impairment of mitochondrial oxidative metabolism may also play a pivotal role in the pathogenesis of PD as demonstrated by the downregulation of genes responsive to peroxisome PGC-1α (Zheng et al., 2010) which, in turn, is the master regulator of mitochondrial biogenesis and function (Sergi et al., 2019). In further support of this, most studies revealed a reduced activity or downregulation of electron transport chain proteins in the brain and in peripheral sensory neurons (Kaur and Bhardwaj, 1998; Chowdhury et al., 2010; Akude et al., 2011; Stančić et al., 2013; Aghanoori et al., 2017). The relationship between hyperglycemia and neuronal mitochondrial dysfunction is rather complex. In the early stages of hyperglycemia, an upregulation of glycolysis and tricarboxylic acid cycling occurs, most likely due to an increase in glucose supply (Thurston et al., 1995; Kaur and Bhardwaj, 1998; Thakran and Baquer, 2003; Price et al., 2006). Nevertheless, these catabolic pathways became impeded as hyperglycemia progresses (Chowdhury et al., 2010; Akude et al., 2011; Hinder et al., 2013; Aghanoori et al., 2017; Zheng et al., 2017). Remarkably, oxidative phosphorylation is impaired at all stages of hyperglycemia and is independent of the up- or down-regulation of glycolysis and Krebs cycle, indicating that mitochondrial dysfunction precedes glycolysis and TCA hindrance (Kaur and Bhardwaj, 1998; Chowdhury et al., 2010; Akude et al., 2011; Stančić et al., 2013; Aghanoori et al., 2017). This supports the theory that hyperglycemia exerts a metabolic insult to mitochondria leading to an impairment in oxidative phosphorylation (Nishikawa et al., 2000; Du et al., 2001; Brownlee, 2005; Tomlinson and Gardiner, 2008; Giacco and Brownlee, 2010). Thus, despite the initial increase in glycolysis and tricarboxylic acid cycling, this is not supported by a parallel increase in mitochondrial oxidative phosphorylation resulting in oxidative stress and lower energy efficiency. Apart from inducing oxidative stress and ROS, themselves contributing to mitochondrial DNA damage and mitochondrial dysfunction, defective mitochondrial oxidative metabolism also compromises ATP synthesis capacity. Neurons heavily rely on oxidative metabolism to efficiently obtain energy for their physiological functions with mitochondria playing a central role in this process. In light of this, mitochondrial dysfunction compromises neuronal energy availability. Particularly, nigrostriatal neurons have a high energy demand which may explain, albeit in part, their increased susceptibility to hyperglycemia (Renaud et al., 2018). Although hyperglycemia represents the more plausible primary driver of mitochondrial dysfunction, the potential involvement of insulin resistance should not be overlooked in consideration of the role of insulin in inducing mitochondrial biogenesis by promoting the SIRT1/PGC1α pathway, as discussed above (Cheng et al., 2010).

### Rerouting Mechanisms: Polyol Pathway and Macromolecules Glycation

Aside from ROS generated through mitochondrial failure, hyperglycemia may trigger the onset of oxidative stress *via* pathways that dwell upstream the glycolytic enzyme GAPDH (Figure 1).

As previously discussed, free glucose is present in the cytosol of neurons and its concentrations rise during hyperglycemia leading to glucotoxicity. Under normal circumstances, hexokinase is mandated to transform available glucose into glucose 6-phosphate. However, due to substrate-mediated inhibition, in hyperglycemic conditions, it can only funnel a fraction of cytosolic glucose toward the glycolytic pathway before becoming saturated. Excess glucose is therefore consumed by other pathways and is constantly replenished by high extracellular levels. The principal compensatory outlets are the polyol pathway and macromolecule glycation which are two key pathways through which glucotoxicity manifests (Tups et al., 2017).

The polyol pathway is driven by aldose reductase whose affinity for glucose is lower compared to hexokinase (Gabbay et al., 1966). Under physiological glucose concentrations, aldose reductase channels only a minimal amount of glucose toward the polyol pathway, with the aldose reductase-dependent conversion of glucose into sorbitol only significantly taking place when intraneuronal glucose concentrations are elevated. Increased processing of glucose through the polyol pathway drains pools of cofactors required for the activity of a plethora of enzymes, including glutathione reductase, GAPDH and others in the glycolytic pathway and TCA (Vander Jagt et al., 1995). The depletion of NADPH contributes to oxidative stress in light of its role as a cofactor for glutathione reductase which is crucial in maintaining the intracellular pool of reduced glutathione, an aspect of paramount importance for neurons considering their susceptibility to oxidative stress (Barnett et al., 1986). More importantly, the depletion of NAD^+^ operated by sorbitol dehydrogenase represents a key pathological mechanism exploited by the polyol pathway. The reduction in the intracellular availability of NAD^+^ leads to an inhibition of GAPDH with the consequent accumulation of the metabolic intermediates upstream of this enzyme, including triose phosphates. The buildup of triose phosphates leads to the synthesis of methylglyoxal a precursor of the AGEs (Figure 1), described below (Tups et al., 2017). The buildup of fructose, both upstream GAPDH as well as a product of the polyol pathway, may represent a further mediator of the neuronal damage induced by hyperglycemia. In keeping with this, fructose 6-phosphate represents the precursor of UDP-N-acetylglucosamine, the end-product of the hexosamine pathway (Figure 1; Brownlee, 2005). Excess UDP-N-acetylglucosamine induces pathological posttranslational modification by promoting aberrant protein glycosylation which compromises proteins’ function and, as a consequence, cellular homeostasis (Brownlee, 2005; Tomlinson and Gardiner, 2008). Nonetheless, despite the increase in intraneuronal glucose levels providing a mechanistic ground for the activation of these pathways, their role in mediating the effect of hyperglycemia in the pathogenesis of PD remains to be fully elucidated.

The second outlet of excess glucose is *via* macromolecule glycation, which may indirectly contribute to oxidative stress (Vicente Miranda et al., 2016). Glycation consists of the slow non-enzymatic, energy-independent, covalent addition of a monosaccharide to macromolecules, usually lipids or proteins; glycated macromolecules are termed AGEs. Although glucose is a poor glycating agent, it can react with free amino groups leading to the formation of a Schiff base which can then undergo an irreversible rearrangement to generate an Amadori product which accumulates in the tissues initiating the process of advanced glycation (Ulrich and Cerami, 2001). Intracellular AGEs are harmful to cells because they induce the loss or alteration of cellular normal protein or lipid functions, which occur despite the cells are empowered with mechanisms aimed at ensuring the prophylactic clearance of glycating agents (Ahmed et al., 2003). Nonetheless, in the setting of hyperglycemia, excess glycating agents may overwhelm these pathways leading to the formation of AGEs, evidenced by the proportional rise in CNS glycation as a function of glycemia, which can reach an exorbitant 34-fold increase (Uchiki et al., 2012). Most importantly, however, glyoxalase employs glutathione to deactivate the AGEs precursor methylglyoxal, while we know that aldose reductase depletes pools of NADPH (Thornalley, 1988; Schieber and Chandel, 2014). Therefore, methylglyoxal and other glycating agents derived from glucose or its metabolites indirectly worsen the oxidative status of neurons by monopolizing endogenous antioxidative defenses or the cofactors necessary for their restoration. Furthermore, AGEs may exacerbate oxidative stress and oxidative stress itself fosters the detrimental effects of AGEs by upregulating the receptor for AGEs (RAGE) *via* NF-κB activation thereby creating a vicious cycle (Nowotny et al., 2015).

AGEs may also occur extracellularly or being directly secreted from the cells in which they are generated to induce oxidative stress and inflammation in a variety of cells (Gao et al., 2017). AGEs trigger oxidative stress by binding their cognate membrane receptor, the RAGE (Stern et al., 2002) and activating downstream mediators know to induce oxidative stress, such as the inducible nitric oxide synthase (iNOS; Nowotny et al., 2015). Nevertheless, despite AGEs being described for their ability to promote oxidative stress in peripheral tissues (Nowotny et al., 2015), their role in oxidative stress in PD remains to be elucidated.

As described for the induction of oxidative stress, the activation of the RAGE by AGEs triggers deleterious inflammatory pathways (Singh et al., 2001; Jakus and Rietbrock, 2004). Binding of RAGEs by AGEs or other endogenous ligands induced by hyperglycemia is a known mechanism of systemic inflammation in DM and can trigger intracellular signaling cascades that lead to the activation of NF-κB (Bierhaus et al., 2005; Rong et al., 2005; Haslbeck et al., 2007). Although in the brain parenchyma neuroinflammation is primarily mediated by glial cells, it can also be triggered by a direct insult of nutrient-overload to neurons (Gao et al., 2017; Sergi et al., 2018a,b), which can exert additional extraneuronal oxidative injuries.

Gathering all the evidence together, hyperglycemia wields its detrimental effects on neurons by an assortment of conceivable mechanisms leading to oxidative stress:

1. The initial rise in glucose metabolism enhances electron donor supply to the mitochondrial electron transport chain, which intensifies the rate of superoxide anion formation. Superoxide anion damages mitochondrial DNA, which may lead to global mitochondrial failure in the long term. Furthermore, superoxide anion exacerbates ROS production that causes nuclear DNA strand breaks, thereby activating PARP1.
2. Depletion of NAD^+^ by PARP1 and the polyol pathway inhibit glycolysis, tricarboxylic acid cycling and oxidative enzymatic reactions operating *via* enzymes that require this cofactor. An intracellular decrease in NAD^+^ induces the inhibition of the glycolytic enzyme GAPDH, which provokes upstream accumulation of metabolites which are then diverted toward the synthesis of toxic intermediates.
3. Accrued levels of triose phosphates upstream of GAPDH are non-enzymatically converted into the glycating agent methylglyoxal whose decomposition by glyoxalase and aldose reductase expends antioxidative cofactors, such as glutathione and NADPH.
4. Excess glucose can be diverted into the deleterious polyol pathway, powered once again by aldose reductase that further depletes stocks of NADPH required for the renewal of the antioxidative cofactor glutathione and by sorbitol dehydrogenase which reduces intracellular NAD^+^.

What emerges here is a number of different pathways that collectively cause a shortage of antioxidant defenses layered with mitochondrial dysfunction, two pivotal factors likely responsible for pushing vulnerable cells beyond the critical coping threshold. The sum of these undesirable processes appears to be particularly relevant in neurons, in light of their relatively low glutathione levels and their reliance on the pentose phosphate pathway (PPP) for its rapid regeneration (Bolaños et al., 1996, 2008; Kang et al., 1999). In fact, insufficient provision of the PPP leads to poor regeneration of glutathione and neuronal apoptosis (Herrero-Mendez et al., 2009). Neurons may reach the coping threshold quicker than other brain cells, for instance, astrocytes that display comparatively greater PPP activity (García-Nogales et al., 2003; Herrero-Mendez et al., 2009).

Most importantly, the proposed molecular events leading to neuronal oxidative stress in hyperglycemia, explicitly *via* increased superoxide anion generation (Ziegler et al., 2015), mitochondrial dysfunction (Patti and Corvera, 2010), PARP1 activation (Obrosova et al., 2005), impairment of the PPP (Ziegler et al., 2017), glycation (Aubert et al., 2014) and glutathione system deficits (Kasznicki et al., 2012; Mendez et al., 2015), are all cogently established in diabetic patients with neuronal pathology. Remarkably, clinical trials employing pharmacological agents to target some of these pathways, including aldose reductase inhibitors to diminish polyol pathway influx (Greene et al., 1999; Obrosova et al., 2002; Kawai et al., 2010; Sekiguchi et al., 2019) or exogenous superoxide dismutase (SOD) to quench superoxide anion (Bertolotto and Massone, 2012), have been demonstrated to improve diabetic peripheral nerve damage and oxidative status, further solidifying the importance of oxidative stress in neuronal affections arising from hyperglycemia in humans. Unquestionably, though, glycemic control remains the most accessible means to preclude oxidative stress-induced diabetic complications in the brain and in peripheral nerves.

## Conclusion

Today, little doubt remains as to the hazard that a diabetic prelude represents in the development of PD. Even if the etiopathogenesis of PD remains largely enigmatic, in part due to our lack of comprehension regarding the seemingly selective vulnerability of the nigrostriatal dopaminergic pathway to undergo degeneration, ascending hypotheses grant a preeminent role to the existence of a particular phenotypic liability that characterizes neurons of the substantia nigra *pars compacta* and that may render them more sensitive to oxidative stress.

Hyperglycemia represents an additional cause of superoxide anion production, mitochondrial dysfunction, antioxidative bankruptcy, ubiquitin-proteasome system failure and dopamine toxicity that may well favor the early demise of nigrostriatal dopaminergic neurons. Thus, considering the deleterious effects of neuronal glucotoxicity, improving glycemic control in diabetic patients should represent a valuable strategy to decrease the risk of developing PD.

## Author Contributions

DS wrote and edited the manuscript and drew the figure. JR wrote the manuscript. NS revised the manuscript. M-GM designed the manuscript, drew the figure and revised the manuscript.

## Conflict of Interest

The authors declare that the research was conducted in the absence of any commercial or financial relationships that could be construed as a potential conflict of interest.
